# Supplementary material for: Sustainable Return to Work for Workers with Mental Health and Musculoskeletal Conditions
Source: Int J Environ Res Public Health. 2023 Jan 6;20(2):1057. doi: 10.3390/ijerph20021057 (PMC9859362; doi:10.3390/ijerph20021057)
Supplement: Supplementary file 1 [file ijerph-20-01057-s001.zip › ijerph-2029526-supplementary.pdf]

Supplementary material:

Sustainable return to work for workers with mental health and musculoskeletal conditions

International Journal of Environmental Research and Public Health

Table S1. Participant description.

RTW Employees	A	DOA	G	HC	JL	MS	O	WHPW	YOS
**Cases\\001-F-40+**	Over 40	11 weeks	Female	MSDs + CMDs	Non-Managerial	Married	Org 1	30	12 years
**Cases\\002-F-30+**	30-40	Unassigned	Female	MSDs + CMDs	Non-Managerial	Married	Org 1	37	5 ½ years
**Cases\\003-M-40+**	Over 40	5 ½ months	Male	CMDs	Non-Managerial	Married	Org 1	37 ½	9 years
**Cases\\004-F-40+**	Over 40	3 months	Female	CMDs	Non-Managerial	Separated	Org 1	37	2 years
**Cases\\005-F-40+**	Over 40	6 months	Female	CMDs	Non-Managerial	Married	Org 2	35	10 years
**Cases\\006-M-40+**	Over 40	14 weeks	Male	MSDs	Non-Managerial	Married	Org 2	37	10 years
**Cases\\007-F-40+**	Over 40	4 months	Female	MSDs	Non-Managerial	Married	Org 1	21hrs 45 mins	26 ½ years
**Cases\\008-F-40+**	Over 40	6 weeks	Female	MSDs	Managerial	Married	Org 1	37 ½	6 years
**Cases\\009-M-40+**	Over 40	7 months	Male	CMDs	Non-Managerial	Married	Org 2	37 ½	10 years
**Cases\\010-F-30**	30-40	4 weeks	Female	CMDs	Non-Managerial	Separated	Org 1	30	4 years
**Cases\\011-F-40+**	Over 40	5 months	Female	CMDs	Managerial	Married	Org 1	37 ½	5 years
**Cases\\012-F-30+**	30-40	6 weeks	Female	MSDs	Non-Managerial	Divorced	Org 2	28	10 years
**Cases\\013-F-40+**	Over 40	10 weeks	Female	MSDs	Non-Managerial	Divorced	Org 1	37 ½	5 years
**Cases\\014-F-40+**	Over 40	2 weeks	Female	MSDs	Non-Managerial	Married	Org 1	37	3 years
**Cases\\015-F-40+**	Over 40	4 months	Female	CMDs	Non-Managerial	Married	Org 1	18 ½	5 years

**Cases\\016-F-40+**	Over 40	9 weeks	Female	MSDs	Non-Managerial	Single	Org 1	37 ½	8 years
**Cases\\017-F-40+**	Over 40	5 weeks	Female	MSDs	Non-Managerial	Single	Org 1	22	5 years
**Cases\\018-M-40+**	Over 40	4 months	Male	CMDs	Managerial	Single/ Cohabiting	Org 1	37	25
**Cases\\019-M-30+**	30-40	6 weeks	Male	CMDs	Non-Managerial	Single/ Cohabiting	Org 1	45	12 years
**Cases\\020-M-40+**	Over 40	2 months	Male	MSDs	Managerial	Married	Org 1	37	9 years
**Cases\\021-F-40+**	Over 40	3 months/ 5 weeks	Female	CMDs	Non-Managerial/Managerial	Married	Org 1	37	4 ½ years
**Cases\\022-M-40+**	Over 40	5 ½ months	Male	CMDs	Managerial	Married	Org 1	37	5 years

A: age, DOA: duration of absence, G: gender, HC: health condition, JL: job level, MS: marital status, O: organisation, WHPW: working hours per week and YOS: years of service.

Table S2. Realist evaluation phases and data sources.

Realist evaluation Phase	Activity	Source of data
**Phase 1 – Theory gleaning**	Theories for this study were first gathered deductively from a thorough review of existing literature reporting on the effects of organisational factors on sustainable return to work, and then inductively from inferences from managers who are responsible for the RTW process. See **Supplementary material 4** for results of the theory gleaning phase.	• Literature review<br>• Informal semi-structured interviews were undertaken with five managers to explore the implementation of the RTW process and what factors influences employee's decisions to return to work or facilitates a sustainable RTW<br>• Semi-structured interviews with 22 participants sick-listed with MSDs and CMDs
**Phase 2 – Theory refining or creation**	In this phase, data were analysed, and theories generated from the data to aid comparison with initial theory.	• Data analysis and interpretation<br>• Refining the context-mechanism-outcome (CMO) configuration<br>• Interviews with the same participants (20) to clarify refined CMO.
**Phase 3 – Theory consolidation**	In this third phase, final theories corroborated by	• Data Analysis and interpretation

	participants in the second interviews, and the theories more worthy of consideration were finally fine-tuned (Manzano, 2016).	• Consolidation of refined CMO configurations.

Table S3. Results of Phase 1 – Theory gleaning.

Theory gleaning approach	Activity	RTW Factor	Interview extract
Systematic review	A systematic review of the RTW literature was conducted to identify the key organisational facilitators of a sustainable RTW for people with CMDs and MSDs.	**Workplace Support**: This is the most consistent evidence in studies investigating facilitating factors for sustainable RTW after a period of ill-health due to CMDs and MSDs (Etuknwa, et al. 2019). It is the reason the role played by line managers particularly is considered an important aspect of the RTW process (Amir, et al., 2010). Evidence suggests that supervisors who communicate positively with returning employees can significantly reduce the duration of disability, while negative contact with these employees is likely to impede the success of the RTW process (McGuire, et al., 2016). According to Author A *et al.* (2019), support from these workplace leaders is effective in boosting returning worker's work attitude and self-efficacy, which in turn impacts RTW outcomes positively.	**N/A**
Interviews with Line managers	Four-line-managers who coordinate the RTW process were interviewed prior to study data collection from sick-listed employees to aid in unpacking key ideas around the RTW process under evaluation (Pawson & Tilley, 2004). The interviews were audio-recorded and	**Good quality RTW**: In the accounts of all managers, a good quality RTW process was identified as a key facilitator of sustainable RTW for employees who have been sick listed by all managers. According to the managers, a phased return is the most commonly implemented work accommodation for employee's returning to work	"*It's just kind of incrementally built up each week and I personally would disperse that with other flexible working options such as working from home, cause I know that one of the things that probably prevents people coming back to work soon … You know, as soon as they could in this*

	transcribed. Data collection was focussed on identifying the main factors underpinning sustainable RTW; as such, a content analysis was undertaken. The content analysis aimed to identify themes that were mentioned by more than two stakeholders. The final analysis, therefore, identified one frequently mentioned organisational-based theme underpinning sustainable RTW: good quality RTW process.	from long-term absence. However, two managers believed that it is more effective when implemented with flexible working options.	*environment is knowing as soon as you come back into the office, it's like you never went away, phased return or not the work is piled up and the work is back at you like a ton of bricks, and so I think that probably keeps people away for longer*"
		Additionally, one manager suggested that a manager's level of understanding on the nature and cause of employee's ill-health plays a big role in the manager's ability to put in place the most effective preventive measures on the employee's return. Suggesting that competence is heightened by having a good understanding of employee's conditions along-side their limitations.	"*I think having a clear and full understanding of the underlined reasons and causes for the problems, whether they are work-related or non-work-related. And there needs to be a fully supported process for particularly the person who is experiencing the problems in order for them to understand and be able to know what the causes in contribution are".*
		**Workload clarity**: Consequently, two managers noted that where clarity on workload is not clearly communicated to employees during the RTW process, it may impact negatively on employees' ability to return sustainably. One manager argued that because fear over workload is a likely barrier to RTW for workers who have been on long-term sickness absence, it is important to reassure these returning workers of the team-based nature of the workload on their return. This reassurance would be effective in assuaging their fears and ease transition back to work, which in turn impacts on a successful return to work.	"*I think it's reassurance of workload for a lot of my team, because they can be worried about their workload. So, it's reassuring them that it's ok, it's not affecting their job and encouraging them to relax. I suppose that is people's biggest fears, they're gonna feel like their letting the team down, just making sure they're realising that you're part of this, you understand what's going on, you're working with them and it's just being empathetic*.

		**Workplace support**: The theme workplace support was also identified in the systematic review as an important factor, thus strengthening suggestions around the role of leaders and co-workers in the workplace in either impeding or facilitating RTW outcomes. However, it is unclear what elements differentiate helpful support from unhelpful support. The accounts of all managers revealed that the dynamics between managers and staff was perceived as a critical facilitator of a successful RTW after ill-health. One manager implied that as a manager, having a good relationship that does not necessarily revolve around work alone with staff influences one's ability to offer the best help and support during the return process.	"*You know, I think having that relationship with the team. I've got a relationship with my team in that I do know what they are doing and what's going on in their lives…….. They've all got very different lives but having that understanding and having that constant communication helps. And I think when you've got managers who are absent from their team, that's when it can be difficult, the relationship isn't there. So, I think the manager's relationship with the staff is really important.*"

Table S4. Topic guide for managers.

1. Do you manage the return-to-work process for sick-listed employees?
2. Explain how the return-to-work process works, and the role of managers.
3. What factors do you suppose are likely to facilitate a successful RTW or impact decisions to RTW for sick-listed employees?
4. What factors are likely to impede a successful RTW for sick-listed employees?
5. Do you think these factors are gender-related?
6. Is there anything else you think is important in aiding sustainable RTW for you that we have not talked about?

Table S5. Initial theories with CMO configuration.

Themes	CMO Initial Theories
**Good quality RTW process**	1. A supportive individual manager who has the relevant skills and knowledge (context), a high level of understanding regarding employee's nature of condition (context), and who is willing to effectively phase employee's return and, also consider other flexible working options/ accommodations to help ease of transition back to work (context), are more likely to successfully implement good quality RTW processes (mechanism) which impacts on sustainable RTW (outcome).
**Workload clarity**	2. Reassuring workers of their workload during the RTW process (context) is effective in assuaging fear (mechanism) and assisting in easy transition back to work (outcome), which in turn impacts on successful RTW (outcome).
**Workplace support**	3. Line-managers who have a good relationship with sick-listed employees are likely to be more supportive of employees during the RTW process (context), boosting returning worker's work attitude and self-efficacy (mechanism), which in turn impacts sustainable RTW outcomes positively (outcome).

Table S6. Topic guide for Employees.

Participant IDNO |__|__|__|__| Gender Male / Female Researcher Initials |__|__|__|

Date |__|__/__|__/__|__|

Introduction

I am ______________________________ from ______________________

- ✓ General purpose of the study
- ✓ Aims of the interview and expected duration
- ✓ Who is involved in the process (other participants)
- ✓ Why the participant's cooperation is important
- ✓ What will happen with the collected information and how the participant/target group will benefit
- ✓ Any questions?
- ✓ Consent

Warm up [demographic & work history]

Can I ask some details about you and your job?

Job Title ____________________________ Job level____________

Years worked at this facility |__|__|years|__|__|months

Educational Background: □ High School □ College □ University

What department do work in? ____________

How many hours/weeks? ____________

How old are you? □ Under 30yrs □ 30-40yrs □ Over 40yrs

Are you married/ Single or cohabiting? □ Yes □ No

Do you have any children? □ Yes □ No

Health condition and duration of absence? ____________

Is it a recurrent condition? ____________

Did you return to full time or part time work? ____________

Now I am going to ask you some questions about your perception about work, the return to work process and certain factors, circumstances or situations that you feel could facilitate a sustainable RTW.

**Domain**	**Topic and Probes**
**Context**<br><br>+	**RTW Process**<br>1. Could you tell me about your views on the return-to-work process?<br>Probes.

**Mechanism**<br><br>+<br><br>**Outcome**	• Do you consider the process helpful/ or not? Why?<br>• What challenges were concerned about you would encounter on returned to work?<br>• How straightforward did you think it was to return to work? What made it easy or difficult?<br>• Is there anything about the RTW process that is most likely to discourage you from returning to work?<br>• How satisfied are you in general with the RTW process?<br>**Work adjustment**<br>2. Could you tell me about how the nature of your job and the work environment affected return to work?<br>Probes.<br>• Was your job different from before absence? How? Did you need any work adjustments? Tell me about that.<br>• How did you perceive your employers/supervisor's willingness to adjust your work? Was the adjustment beneficial?<br>**Workplace encounter/ support**<br>3. Could describe your relationship with your 1st manager and 2nd colleagues at work, how would you qualify their role in your return to work? Supportive or not?<br>Probes.<br>• What were the specific expectations you had with regards to the level of support you expected from them on your RTW? By that I mean, what did you expect them to do to show support? And do you think they met it?<br>• Do you think knowing that you have a supportive team or line manager could motivate you to return to work?
**Closing**<br>Is there anything else you think is important in aiding sustainable RTW for you that we have not talked about?<br>✓ Summarise<br>✓ Thank participant<br>✓ Provide extra information and contacts to participants	

Table S7. Table showing the consolidation method.

Initial CMO configurations	Exemplar quotes	Theoretical abstraction
A competent and supportive manager, working in collaboration with other health services within the organisation (context) is likely to increase their level of understanding about employee's condition and best RTW approach to adopt, as well as be more empathic towards employees (mechanism). As a result, they can successfully implement an effective RTW strategy (mechanism) approved by senior management, thus impacting on sustainable RTW (outcome)	*"I think it obviously depends on what your managers have to deal with if you like, ...But I think because they have the Occupational Health and the HR and their guidance and obviously the HR team and the Wellbeing team would have dealt with a lot more situations with people's mental health situations. I think they're supported by the other members of the organisation, so they are able to support you. Even if they might not understand your situation that they haven't dealt with any mental health issues themselves, I think they're supported enough that they can be empathetic" (021-F-40+).*<br><br>*"Well, a combination of the two really. It was occupational health who were obviously involved and then the colleagues that's just sort of a subcontracted situation. But they, if I recall correctly ... I went and spoke to them a couple of times and they made recommendations around the phase return and then my*	It is the choices LMs are presented with as well as their motivation to be supportive<br><br>When managers work in collaboration with contracted support services, they have a better understanding of employee's condition and its wider impact, they are more empathic towards sick-listed employee, and are better equipped to provide the most appropriate RTW strategy

	manager then took that on board. It was phased in terms of numbers of days of the week (009-M-40+).”<br><br>*I think just… the main thing is having a supportive line manager and then an understanding one. And someone who wants to work with you to help you get back to work and so you’re not made to feel guilty, cause you’re already feeling guilty enough yourself anyways Uhm… without having any extra pressure. Because that one little thing is sometimes enough to stop someone from making that next step. (004-F-40+)*<br><br>*“Uhm. I think all line managers are a practice consultant, and practice consultants depend… each is a bit different, and I think that they do try their best, but they are blocked from the service managers cause service managers will sign, ‘we can’t do this’, ‘we can do this. I think it was blocked from the service manager level. So, the practice consultant had no say or control. And to be fair it’s only the service managers who have the power, the practice*	

	consultants don't. So, they just have to go through the flow. They have to go and ask the right question and if we do say 'could we try this?' like 'Could we work from home?' 'What about this?'... They can't say yes or no. It has to go to the service managers and then it's blocked at the service manager level. So, there's not really much concession." (002-F-30+)	
When employees sick listed with CMD return to toxic working environments (context) during the RTW process (mechanism), it is likely to aggravate their condition, leading to a failed RTW (outcome).	"*It's difficult because as I said then, going back to this previous line manager and you know after I got back last time and then dealing with this person who I could have put in a complaint with. So, it's basically being line-managed by the person who was the problem and so I suppose it was an unusual case in that respect.*" (019-M-30+, CMDs)<br><br>*"I think I got stronger, so my energy came back and the situation between my supervisor and my manager hasn't really improved. There should be a mediation process which took quite a bit of time and it's still not started. However, my situation is*	Ongoing process of renegotiation workplace accommodations including workload - factors hindering both employees and manager choices.

	such that I look… if I find something different without putting me under pressure. We have a Wellbeing service and coming back they should have done the mediation between my manager, my supervisor and me, but they are quite overwhelmed with work so they can't really do that. So that's… at the moment where I am." (015-F-40+)<br><br>*"Basically, the fear was I just felt I wanted to keep a job, so, I just agreed to it. Even though it wasn't the best for me, it was probably the best for the service, so to speak. But I did obviously keep an eye out for other opportunities as they came up." (022-M-40+)*<br><br>*"There's not a lot of support. It's that fine balance, because if I did start to go and say 'I need help, I think you should do something for me…' I worry would that then penalise me and then I start to go on a 'well, she's not fit for work, get her gone'. So, it's really tricky to know how much do you say you're struggling and how much don't you? So, I just go on with it,*	

	I do the job as best as I can....” (002-F-30+)	
Reassuring workers of their workload during the RTW process (context) is effective in assuaging fear (mechanism) and assisting in easy transition back to work (outcome), which in turn impacts on successful RTW (outcome).	*“I think it could be better simply by better communication and getting a clearer picture of what somebody can do when they come back rather than you go to occupational health, they say phased return, so your manager sits down with you, and you work out the pattern of phased return and then off you go.*” (016-F-40+)<br><br>*“... when they are talking about phased return, I thought my understanding was it was just about number of days. You know you start with two and have a break. But it was more the task. So my recommendation is that it's clear to people around task and time.”(011-F-40+)*<br><br>*“Well, I think coming in a little bit flexibly really helped, because I was able to sort just work reduced hours. So, you know, just coming in and going... so if I found myself a little bit tired or stressed then I was able to take a little break and that helped quite well. Just having that flexibility... you know people*	Ongoing process of renegotiation workplace accommodations including workload.<br>On-going negotiation: where conversations around workload are raised, challenges posed by individuals that impede their current state of recovery can accommodate on RTW could be easily assuaged

	were there for me to sort of seek advice from" (003-M-40+)	

Table S8. Classification of sickness absence period and RTW strategy.

**RTW Employees**	**Duration of Absence**	**Health Condition**	**RTW Strategy**	**Class of absence**
**Cases\\001-F-40+**	11 weeks	MSDs + CMDs	Phased Return	Long-term
**Cases\\002-F-30+**	5 weeks	MSDs + CMDs	Flexible working options	Short-term
**Cases\\003-M-40+**	5 ½ months	CMDs	Phased Return	Long-term
**Cases\\004-F-40+**	3 months	CMDs	Phased Return	Long-term
**Cases\\005-F-40+**	6 months	CMDs	Phased Return	Long-term
**Cases\\006-M-40+**	14 weeks	MSDs	Phased Return	Long-term
**Cases\\007-F-40+**	4 months	MSDs	Phased Return	Long-term
**Cases\\008-F-40+**	6 weeks	MSDs	Flexible working options	Short-term
**Cases\\009-M-40+**	7 months	CMDs	Phased Return	Long-term
**Cases\\010-F-30**	4 weeks	CMDs	Flexible working options	Short-term
**Cases\\011-F-40+**	5 months	CMDs	Phased Return	Long-term
**Cases\\012-F-30+**	6 weeks	MSDs	Flexible working options	Short-term
**Cases\\013-F-40+**	10 weeks	MSDs	Phased Return	Long-term
**Cases\\014-F-40+**	2 weeks	MSDs	Flexible working options	Short-term
**Cases\\015-F-40+**	4 months	CMDs	Phased Return	Long-term
**Cases\\016-F-40+**	9 weeks	MSDs	Phased Return	Long-term
**Cases\\017-F-40+**	5 weeks	MSDs	Flexible working options	Short-term
**Cases\\018-M-40+**	4 months	CMDs	Phased Return	Long-term
**Cases\\019-M-30+**	6 weeks	CMDs	Flexible working options	Short-term
**Cases\\020-M-40+**	2 months	MSDs	Phased Return	Long-term
**Cases\\021-F-40+**	3 months/ 5 weeks	CMDs	Phased Return	Long-term
**Cases\\022-M-40+**	5 ½ months	CMDs	Phased Return	Long-term
